# Characterization of the complete chloroplast genome of the medicinal herb *Veronica polita* Fr. 1819 (Lamiales: Plantaginaceae)

**DOI:** 10.1080/23802359.2022.2086074

**Published:** 2022-06-30

**Authors:** Shouning Jia, Wenjuan Chen, Guofu Zhao, Shuangxi Wang, Zhiwei Xu, Qien Li

**Affiliations:** a Qinghai Provincial Hospital of Traditional Chinese Medicine, Xining, People’s Republic of China; b Tibetan Medicine Research Center, Tibetan Medical College, Qinghai University, Xining, People’s Republic of China

**Keywords:** Chloroplast genome, high-throughput sequencing, medicinal herb, phylogenetic analysis, *Veronica polita* Fr. 1819

## Abstract

*Veronica polita* Fr. 1819 (synonym: *Veronica didyma* Ten. 1981) is a species of annual herb with high medicinal value. It is originally from Southwest Asia, but has been naturalized widely in many regions of the world. In this study, the complete chloroplast genome of *V. polita* was determined to be 150,191 bp long with a typical quadripartite structure, comprising two inverted repeat regions (IRa and IRb, 25,465 bp each), a large single-copy (LSC) region (81,847 bp) and a small single-copy (SSC) region (17,414 bp). It encodes a panel of 114 genes (including 79 protein-coding, 31 tRNA, and four rRNA genes) with 18 of them being completely or partially duplicated and 19 of them possessing one or two introns. Phylogenetic analysis supported the tribal-level taxonomy of the family Plantaginaceae, and revealed that *V. polita* was most closely related to the congener *Veronica persica* Poir. 1808.

*Veronica polita* Fr. 1819 (synonym: *Veronica didyma* Ten. 1981) is a species of annual herb in the family Plantaginaceae (order Lamiales) (Schoch et al. 2020). It is originally from Southwest Asia, but has been naturalized widely in the temperate and subtropical regions of the world (Hong and Fischer 1998; Shanghai Academy of Science & Technology 1999). This herb has long been used in traditional Chinese medicine for treating such illnesses as dysmenorrhea, metrorrhagia, and malaria (Nanjing University of Chinese Medicine 2005), and has also recently been reported for anti-inflammatory and antioxidant activities (Akanda et al. 2018). To date, previous studies of *V. polita* have mainly focused upon its chemical composition (Wang et al. 1995), agricultural implications (Li 2003; Zhang J et al. 2006; Zhong and Wang 2010), economic values (Yang et al. 2006), ecology (Tang et al. 2006), physiology (Zhang Y et al. 2010; Li and Xu 2015), and pharmacological effects (Akanda et al. 2018). Its genetics and genomics have yet to be investigated. In this study, the complete chloroplast (cp) genome of *V. polita* was retrieved from Illumina sequencing reads, and its phylogenetic placement was investigated.

Leaf tissues were collected from an individual of *V. polita* in Haihu New District, Xining, Qinghai Province, China (101.73°E, 36.64°N), and were used for the genomic DNA isolation with the DNeasy Plant Mini Kit (Qiagen, Valencia, CA). A specimen was deposited at the Herbarium of Qinghai Provincial Hospital of Traditional Chinese Medicine (http://www.qhzyy.com.cn/; Shouning Jia, Email: iashouning@163.com) under the voucher number VDIDY20210930. High-throughput DNA sequencing was conducted on the Illumina HiSeq X Ten Sequencing System (Illumina, San Diego, CA), yielding 17.88 M of 150-bp paired-end reads in all. The cp genome was assembled using the software MITObim v1.9 (Hahn et al. 2013) with that of *Veronica persica* Poir. 1808 (GenBank accession: KT724052) (Choi et al. 2016) as the initial reference. Annotation of the cp genome was done in Geneious Prime (Biomatters Ltd., Auckland, New Zealand) by aligning with those of its congeners.

The cp genome of *V. polita* is 150,191 bp long with a typical quadripartite structure, comprising two inverted repeat regions (IRa and IRb, 25,465 bp each), a large single-copy (LSC) region (81,847 bp) and a small single-copy (SSC) region (17,414 bp). The base composition is asymmetric (30.7%A, 19.2%C, 18.7%G, and 31.4%T) with an overall A + T content of 62.1% ('light strand'). The A + T contents of LSC (64.1%) and SSC (68.4%) are obviously higher than that of IR regions (56.8%). The cp genome encodes a total of 114 genes, including 79 protein-coding gene (PCG), 31 tRNA, and four rRNA genes. In all, 18 genes are completely or partially duplicated, including seven PCGs (*ndh*B, *rpl*2, *rpl*23, *rps*7, *rps*12, *ycf*1, and *ycf*2), seven tRNA (*trn*A-UGC, *trn*I-CAU, *trn*I-GAU, *trn*L-CAA, *trn*N-GUU, *trn*R-ACG, and *trn*V-GAC), and all four rRNA genes (*rrn*4.5, *rrn*5, *rrn*16, and *rrn*23). Ten PCGs (*atp*F, *ndh*A, *ndh*B, *pet*B, *pet*D, *rpl*2, *rpl*16, *rpo*C1, *rps*12, and *rps*16) and seven tRNA genes (*trn*A-UGC, *trn*G-UCC, *trn*I-GAU, *trn*K-UUU, *trn*L-UAA, *trn*L-UAG, and *trn*V-UAC) harbor a single intron, and double introns occur in a couple of PCGs (*clp*P and *ycf*3). In addition, four microsatellite loci ((A)_16_, (A)_17_, (AT)_7_, and (TTTTG)_3_) were detected in the cp genome using SciRoKo v3.4 (Kofler et al. 2007) with default parameters.

Phylogenetic analysis was conducted based on the Bayesian analysis of cp PCGs for a panel of 28 taxa within the family Plantaginaceae using MrBayes v3.1.1 (Huelsenbeck and Ronquist 2001; Ronquist and Huelsenbeck 2003) (Figure 1). As suggested by the ‘Model Selection’ function of TOPALi v2.5 (Milne et al. 2009), <GTR + G+I > was implemented as the best-fit nucleotide substitution. The outgroup taxa used in this study were three species from the family Scrophulariaceae (order Lamiales), i.e. *Buddleja sessilifolia* B.S. Sun ex S.Y. Bao 1983 (MH411149) (Ge et al. 2018), *Myoporum laetum* G. Forst. 1786 (MN044641) (Fowler et al. 2020), and *Scrophularia buergeriana* Miq. 1865 (KP718626) (Yi and Kim 2016). The phylogenetic analysis appears to support the tribal-level taxonomy of the family Plantaginaceae. The 11 taxa belonging to the tribe Veroniceae were clustered together, and *V. polita* was found to be most closely related to the congener *V. persica*.

**Figure 1.**
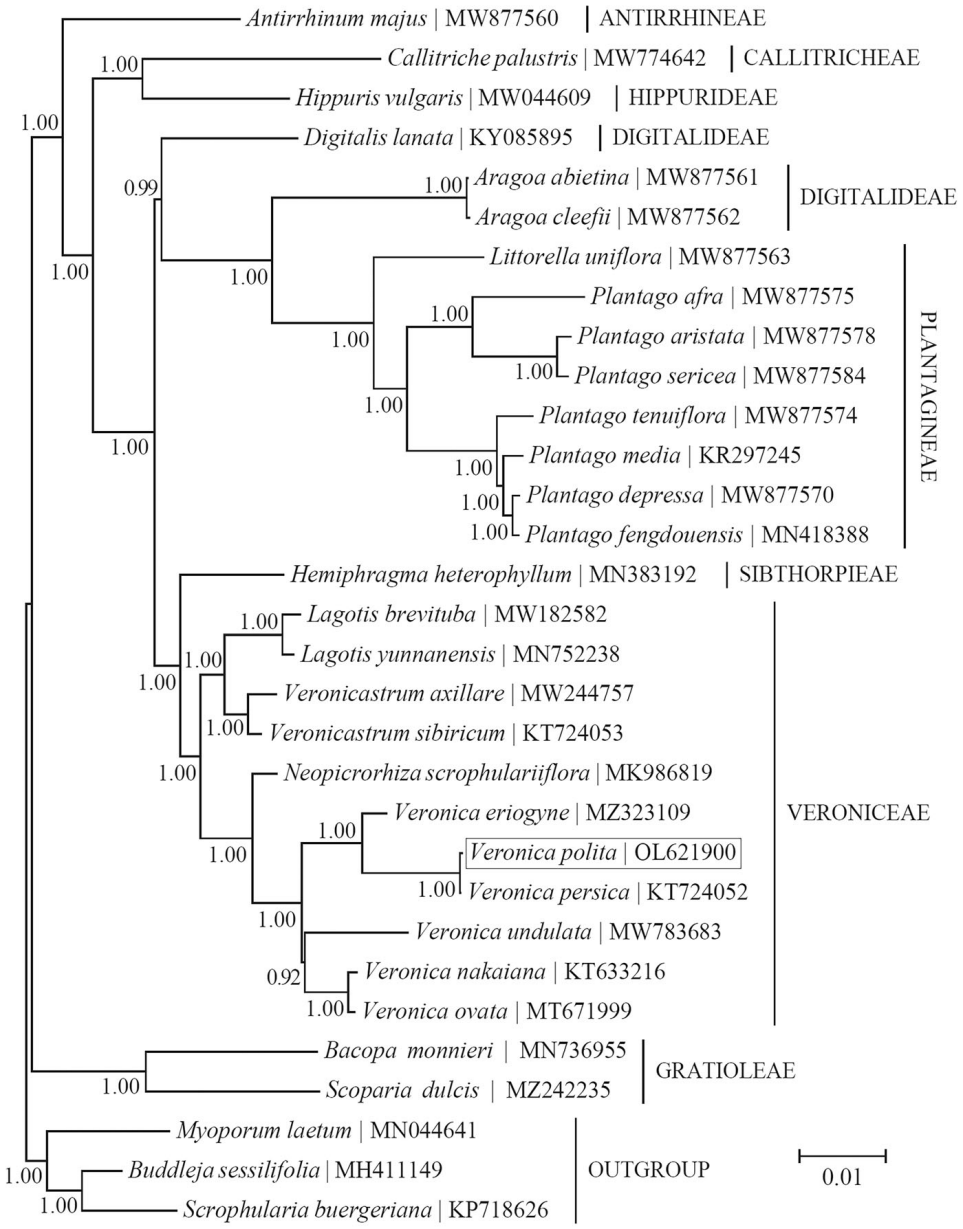
Phylogeny of the family Plantaginaceae based on the Bayesian analysis of the concatenated coding sequences of chloroplast PCGs. The best-fit nucleotide substitution model is ‘GTR + G+I’. The support values next to the nodes were Bayesian posterior probabilities according to the Bayesian analysis. The outgroup taxa included were three species from the family Scrophulariaceae (order Lamiales). Tribal-level taxonomy was presented for each taxon in capital letters.

## Ethical approval

The materials used in this study are not included in the IUCN Red List of Threatened Species or the List of State-protected Plant Species, and the sampling site is not located in any protected area. The field study and laboratory study were conducted in accordance with guidelines provided by Qinghai Provincial Hospital of Traditional Chinese Medicine and Qinghai University.

## Author contributions

Zhiwei Xu and Qien Li were involved in the conception and design; Shouning Jia, Wenjuan Chen, Guofu Zhao, and Wangshuang Xi analyzed and interpreted the data; Shouning Jia drafted the paper; Shouning Jia, Zhiwei Xu, and Qien Li revised it critically for intellectual content. All authors approved the final version to be published, and agreed to be accountable for all aspects of the work.

## Data Availability

The genome sequence data that support the findings of this study are openly available in GenBank of NCBI at https://www.ncbi.nlm.nih.gov under the accession number OL621900. The associated BioProject, SRA, and Bio-Sample numbers are PRJNA689716, SRR17062764, and SAMN23489229, respectively. The annotated chloroplast genome is also available in Zenodo at https://doi.org/10.5281/zenodo.5746098.
